# The Cut-Off Score of Four Clinical Tests to Quantify Balance Impairment in Individuals with Chronic Ankle Instability

**DOI:** 10.21315/mjms2021.28.4.9

**Published:** 2021-08-26

**Authors:** Nontawit Udompanich, Kanok-on Thanasootr, Raoyrin Chanavirut, Uraiwan Chatchawan, Torkamol Hunsawong

**Affiliations:** School of Physical Therapy, Faculty of Associated Medical Sciences, Khon Kaen University, Thailand

**Keywords:** ankle injury, ankle sprains, joint instability, posture, postural controls

## Abstract

**Background:**

Balance impairment is a common consequence of chronic ankle instability (CAI). This study aimed to assess the discriminative validity of four clinical tests for quantifying balance impairment in individuals with CAI.

**Methods:**

Participants were screened for their balance using the single-leg balance test (SLBT) and were assigned to either the positive or the negative SLBT groups. Fifty-four individuals with CAI (*N* = 27 per group) were recruited and completed four clinical tests including the foot-lift test (FLT), the time-in-balance test (TIBT), the modified star excursion balance test in the posteromedial (mSEBT-PM) direction and the side-hop test (SHT). The receiver operating characteristics (ROC) curve coupled with Youden index were calculated to determine the optimal cut-off scores of each test.

**Results:**

We found significant differences in balance between groups for all tests, with good to excellent values for the area under the ROC curve (AUC). All four tests reached good to excellent sensitivity and specificity values and had significant cut-off scores to discriminate balance performance among CAI participants.

**Conclusion:**

All four clinical tests can be conducted with their respective cut-off scores to quantify balance impairment in individuals with CAI.

## Introduction

A lateral ankle sprain (LAS) is a major injury that frequently occurs in athletes and physically active populations, and it has the highest recurrence rate of all musculoskeletal injuries (1). Notably, over 30% of individuals with an index LAS will suffer from long-term symptoms caused by repeated injuries, including recurrent LAS, pain, limited ankle joint range of motion and the development of chronic ankle instability (CAI) (1–2). Individuals with CAI generally experience episodes of giving way and a feeling of ankle joint instability (3–4). Typically, balance impairment is addressed as a result of sensorimotor deficits related to CAI that occurs after damage to the lateral ligament complex (1, 3, 5, 6).

Within 12 months of the initial injury, some individuals with CAI (i.e. copers) demonstrate successful sensorimotor adaptation and display no further symptoms (7). In contrast, those who cannot improve their sensorimotor function during this period will likely sustain balance deficits and re-sprain their ankles (7–8). Thus, individuals with CAI benefit from tools that monitor and detect balance improvements. A previous meta-analysis suggested that force plates and non-instrument clinical balance tests are useful for identifying balance impairment in individuals with CAI (5). Although the force plate is a valid and accurate tool for quantifying balance (5), it is expensive and might not be available to a given athlete. Therefore, non-instrument clinical tests suitable for various settings are important for assessing individuals with CAI.

The single-leg balance test (SLBT) is a clinical screening method for balance impairment (9). The SLBT has excellent inter-rater agreement and can efficiently predict LAS in athletes (9). Although this test is easy to apply, it may not appropriately monitor impairment progression or determine balance recovery, as it delivers only positive-negative results. Consequently, numerous quantitative tests have been developed to estimate static or dynamic balance performance in individuals with CAI (6, 10), including the foot-lift test (FLT), the time-in-balance test (TIBT), the modified star excursion balance test in the posteromedial (mSEBT-PM) direction and the side-hop test (SHT).

The FLT is a commonly used static balance test that requires individuals to maintain 30 sec of single-leg balance and tracks the number of foot lifts and errors (10–11). Likewise, the TIBT is another static balance test and it evaluates the time in seconds that individuals can stand on the CAI limb with their eyes closed (12), resulting in a large effect size between CAI and control groups (5). In contrast, the mSEBT-PM assesses dynamic balance and neuromuscular control, and this variation applies the most representative direction for identifying CAI (13). Individuals perform unilateral squats on the tested limb while reaching as far as possible with the contralateral limb (10). Individuals with CAI have worse scores when using their CAI limb on the mSEBT-PM compared to their uninjured limb and control subjects (10, 13). Lastly, the SHT is another measure of dynamic balance and it requires agility, as individuals must hop and land in a side-to-side fashion. The test movement also challenges ankle joint stabilisers and simulates the LAS mechanism (10, 14).

According to the current evidence, balance impairment is more commonly found in individuals with CAI than in control or coper individuals. However, the cut-off scores used to quantify the severity of balance impairment among the CAI population are still limited. Crucially, accurate cut-off scores provide clinical information that may help healthcare professionals and athletic trainers to identify CAI individuals with severe balance impairment and high risks of recurrent injury as well as potential copers. In this study, we examined the discriminative validity of the FLT, the TIBT, the mSEBT-PM and the SHT in CAI subjects. We screened their balance using the SLBT to divide them into positive and negative SLBT groups and then compared four balance outcomes. Finally, we established cut-off scores determining balance among the CAI group.

## Methods

A matched-pair case-control study was launched with physically active individuals and university athletes with CAI. The sample size was calculated using a formula that compares the means of two groups (15) and 27 participants were required for each group. This study was approved by a local institution’s ethics committee. Before data collection, all participants gave their written informed consent.

### Participants

We recruited 54 CAI participants with an age range of 18 years old–40 years old, mainly from university sport clubs. All participants were amateur, physically active athletes and met the following inclusion criteria (4): i) a history of at least one LAS with inflammatory symptoms (pain and/or swelling) that impacted at least one day of physical activity; ii) their first LAS at least 12 months before recruitment; iii) their latest LAS at least 3 months before recruitment; iv) a history of giving way and/or a feeling of ankle joint instability, with at least two episodes within the past 6 months and (5) scores ≤ 25 on the Cumberland ankle instability tool (CAIT) (16). The CAIT is a self-reported questionnaire used to identify individuals with CAI. It has demonstrated good validity (16) and excellent test-retest reliability (ICC_2,1_ = 0.96) (17), and it has also been recommended as a minimum diagnostic criterion (4).

We excluded participants with any of the following conditions: i) a history of previous surgeries and/or any previous fractures in the musculoskeletal structures of the lower limb and/or spine; ii) an acute injury within the past 3 months in any musculoskeletal structure of the lower limb and/or spine; iii) any diagnosed visual disorders without correction; iv) any diagnosed vestibular disorders (10); v) a history of spinal cord or brain injuries or vi) the use of medications such as painkillers and/or anti-inflammatory drugs within 72 h before data collection.

### Procedures

All participants performed the SLBT, FLT, TIBT, mSEBT-PM and SHT. We used the SLBT to screen for balance impairments (10). Following the results, we sorted the participants into positive (case) and negative (control) SLBT groups. Participants in both groups were matched based on gender, age range (18 years old–29 years old and 30 years old–40 years old), weight (±15 kg), height (±10 cm) and CAI limb (10).

All participants performed the SLBT first and then the other tests in a random order that we determined with simple random sampling. All tests were performed on a CAI limb. We assessed the most recent LAS limb in participants with bilateral CAI (10). We asked all participants to refrain from vigorous activities for at least 24 h prior to data collection.

The participants performed all tests except the mSEBT-PM without any practice sessions and they had a 1-min rest between the different tests (6). A rater blinded to the participants’ groups conducted all tests. This rater was an experienced physiotherapist who had completed one year of intensive training in clinical balance tests for athletes. The intra-rater reliability assessment was conducted in physically active individuals and the rater showed a good to excellent level of reliability in all tests (ICCs = 0.76–0.91).

### Single-Leg Balance Test

The participants began by balancing barefoot on a stable surface with the testing limb. They placed both of their hands on their hips and slightly bent their opposite hip and knee without allowing their foot to touch the ground or the testing limb. Next, the participants looked straight ahead, fixed their eyes on the wall, and then closed their eyes for 10 sec (9). The researcher looked for signs of balance impairment and recorded a positive result if they observed any of those signs, including the opposite foot touching the testing limb or the floor, any changes in the base of support and their hands moving from their hips. This test was performed with a 30-sec rest between the two trials (9). If participants had positive results for both trials, they were assigned to the positive SLBT group (9).

### Foot-Lift Test

The participants assumed a single-leg stance by raising their opposite foot. The researcher then instructed them to touch the testing leg with it at the mid-calf level and monitored the number of foot lifts (10–11). The participants remained in this single-leg stance with their eyes closed for 30 sec per trial. This test was performed with a 1-min rest between each of the three trials (6). A foot lift was counted when any part of the testing foot lifted from the floor or the opposite foot touched the floor (10–11). The average value of the three trials was used as the outcome (10).

### Time-In-Balance Test

The participants maintained a single-leg stance using the same position as the SLBT (10) and the researcher timed them while they remained in this stance with their eyes closed. The researcher stopped recording if participants lost their balance (including changes in the base of support), touched the testing limb and/or the floor with the opposite foot or moved their hands from their hips (10). The test was performed with a 1-min rest between each of the three trials, and the maximum time for each trial was 60 sec (10, 12). The longest time of the three trials was used as the outcome (10).

### Modified Star Excursion Balance Test in the Posteromedial Direction

The participants placed their testing foot on the centre of a tape marker and their hands on their hips. The researcher then asked the participants to reach with their opposite leg as far as possible along the tape line, which extended 45° from the centre of the tape marker in the posteromedial direction (10, 13, 18). The participants used the tip of their great toe to touch the tape line, ideally without losing their balance or making any changes to the base of support. The researcher recorded the reach distance (cm) and then divided it by the length of the opposite leg, which the researcher measured with a tape measure from the anterior superior iliac spine to the most prominent part of the medial malleolus (10). The participants were asked to perform six practice trials and rest between them for 10 sec (10). After the practice session, the participants were given a 2-min rest. Afterwards, they performed three trials with a 10-sec rest between each trial. The average value of these trials was used as the outcome (10).

### Side-Hop Test

The participants were instructed to hop barefoot on the testing limb 10 times per trial in the mediolateral direction for 30 cm (14, 19). The total time in seconds needed to complete each trial was recorded. The test was performed with a 1-min rest between the two trials, and the lowest time was used as the outcome (6, 14). If participants stopped hopping prematurely during the testing session, the researcher continued to record the time and encouraged them to continue.

### Statistical Analysis

We used the SPSS programme (version 23.0; SPSS Inc., Chicago, IL) for data analysis. To compare the balance performance results of the positive and negative SLBT groups, we conducted an independent sample *t*-test (20). To determine the differences in magnitude between the groups, we calculated their effect sizes using Cohen’s *d*: *d* = (*μ*_1_–*μ*_2_)/σ (21). We interpreted the *d*-values as follows: ≥ 0.8, 0.5–0.79 and 0.2–0.49 for large, medium and small effect sizes, respectively (21).

For our analysis, we also used the values of the receiver operating characteristics (ROC) curve, which represents the relationship between sensitivity and specificity, and the area under the ROC curve (AUC). These values indicated the discriminative performance of each test as well as their asymptotic significance (α = 0.05) (22). We interpreted the AUC values: 0.9–1.0 as excellent, 0.80–0.89 as good, 0.70–0.79 as acceptable, 0.60–0.69 as poor and 0.00–0.59 as failure (22). We selected the optimal cut-off points based on the largest Youden index (*J* values) throughout the ROC curve, which we calculated as follows: *J* = ([sensitivity+specificity]−1) × 100 (22–23).

We determined the positive and negative likelihood ratios (LR) using the sensitivity and specificity at select cut-off points: LR+ = (sensitivity)/(1-specificity) and LR− = (1−sensitivity)/(specificity) (24). The positive likelihood ratio ranged from zero to infinity. Zero means that a test’s positive result does not identify a true balance impairment and infinity means that a test’s positive result does identify a true balance impairment (24). The negative likelihood ratio ranged from 0 to 1. Zero means that a test’s negative result is a true negative, and 1 means that a test’s negative result is not a true negative (24).

## Results

### Characteristics of the Participants

Fifty-four individuals with CAI (27 per group consisting of 20 males and 7 females) voluntarily participated in the study. The average ages were 23.29 years old and 22.62 years old in the negative SLBT and positive SLBT groups, respectively. No group differences were demonstrated for demographic data, histories of LAS, giving way and/or feeling of ankle joint instability, except for participants’ CAIT scores (Table 1). The negative SLBT group had significantly higher CAIT scores for the CAI limb than the positive SLBT group (*P* = 0.015).

### Differences in Balance Performance

Differences between groups were demonstrated for all balance tests (*P* < 0.001) coupled with large effect sizes (*d* > 0.80). Specifically, the negative SLBT group had superior balance results for all tests (Table 2). For the FLT, the negative SLBT group had a significantly lower number of foot lifts than those in the positive SLBT group (mean difference = −5.95 times). For the TIBT, the negative SLBT group showed a significantly longer time of standing on the CAI limb than those in the positive SLBT group (mean difference = 32.24 sec). For the mSEBT-PM, the negative SLBT group demonstrated a significantly greater normalised reaching distance when compared to the positive SLBT group (mean difference = 9%). Similarly, for the SHT, the negative SLBT group demonstrated a significantly shorter time to complete the test than those in the positive SLBT group (mean difference = −9.48 sec).

### Discriminative Validity

**Figure 1:**
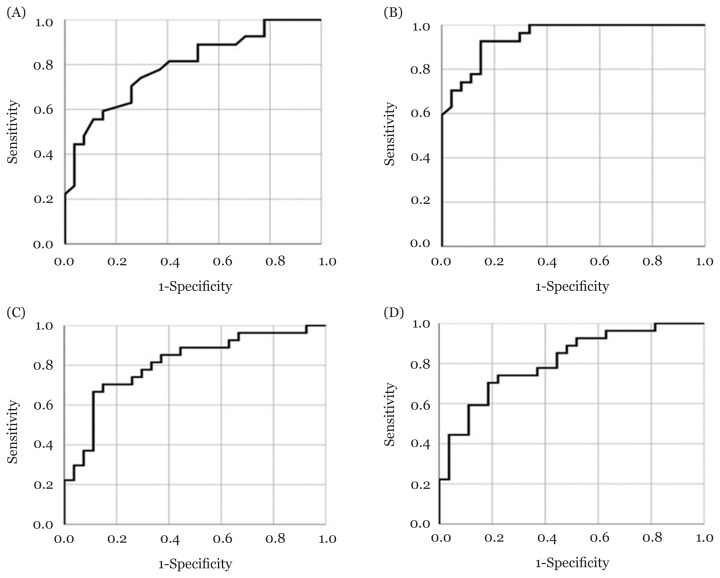
ROC curve plot for (A) FLT, (B) TOBT, (C) mSEBT-PM and (D) SHT

The ROC curves for all four tests are shown in Figure 1. All tests demonstrated excellent AUC values (≥ 0.80) with asymptotic significance (*P* < 0.001). This suggests that all tests had high diagnostic sensitivity and were able to quantify differences in balance performance among individuals with CAI. Table 3 represents the established significant cut-off scores for the FLT (≥ 9.5), TIBT (≥ 30.40 sec), mSEBT-PM (≤ 91.05%) and SHT (≥ 12.85 sec) as well as the sensitivity, specificity, Youden index and positive and negative likelihood ratios that were calculated for each cut-off score.

## Discussion

This study provided diagnostic values for four balance measures used in CAI populations. Regarding the FLT, we determined that those individuals with CAI who lifted their foot at least 10 times had severe balance impairment. Similarly, Linens et al. (10) found a statistically significant difference in the number of foot lifts between CAI and control groups (*n* = 17 per group), with a large effect size (*d* = 0.94) (10). Their cut-off score to discriminate CAI was 5 foot lifts (AUC value = 0.76) (10). Likewise, Hiller et al. (11) performed the FLT with 61 subjects: 20 as external control, 19 with unilateral CAI and 22 with bilateral CAI. They found a statistically significant difference between the numbers of foot lifts made by the CAI and control groups (11). However, they did not report effect sizes or cut-off scores (11).

For the TIBT, we claimed that individuals with CAI who were unable to stand on the affected limbs with their eyes closed for 30 sec had severe balance impairment. Specifically, we found that the TIBT outperformed all other tests in quantifying balance. This finding is in line with that of a previous meta-analysis (5). It reported that the time variable had the largest mean difference among the other measurement units (e.g. velocity, linear and area) (5). Linens et al. (10) found a difference between the TIBT results of CAI and control groups, with a large effect size (*d* = 0.92). Their cut-off score was 25.89 sec (AUC value = 0.73) (10). Chrintz et al. (12) studied 29 CAI subjects and found that they demonstrated worse performance on the TIBT using the injured limb with their eyes open or closed when compared to the uninjured limb and control group (12). Additionally, a meta-analysis by Rosen et al. (24) found a large, pooled effect size (Hedges’ *g* = 0.898) for the TIBT results of CAI and control groups.

To maintain static balance, individuals need proprioceptors that can transfer joint sense to the higher somatosensory area. The higher brain then fires a command to the targeted muscle of the lower extremities, particularly those muscles at the ankle joint, like the soleus and fibularis muscles, which help maintain a quiet stance (10, 25). These ankle strategies are crucial for maintaining the centre of gravity in the base of support (10, 25). A previous study reported that individuals with CAI experienced a sensory deficit at the joint of the injured ankle, meaning that they relied more on visual input and used their proprioceptive senses less than with the normal ankle (25–26). Thus, the present cutoff scores of the FLT and TIBT could be used to monitor changes in static balance performance in individuals with CAI after they have received an intervention that targets the ankle joint’s somatosensory domain.

For the mSEBT-PM, we concluded that individuals with CAI who reached their leg less than 91% of their normalised reaching distance had severe balance impairment and were more likely to be at risk of lower extremity reinjury (27). Plante and Wikstrom (28) compared SEBT results between CAI, coper and control groups. Regarding the normalised reaching distance, they found large differences between the CAI and control groups (*d* = 0.75) and even the CAI and coper groups (*d* = 0.95) (28). Our result is also consistent with that of Linens et al. (10). They compared the SEBT between CAI and control groups using the anteromedial, medial and PM directions. For the PM direction, they found a moderate effect size (*d* = 0.66), a similar cut-off score (91.00% of the normalised reaching distance; AUC value = 0.71) and the largest AUC value of all tested directions (10). Rosen et al. (24) also reported a difference in the mSEBT-PM results between CAI and control groups, with a moderate pooled effect size (Hedges’ *g* = 0.406) (24).

For the SHT, individuals in the negative SLBT group hopped faster than their counterparts. We asserted that individuals with CAI who were unable to complete 10 hops within 13 sec had severe balance impairment. Linens et al. (10) compared balance between CAI and control groups using the SHT, with a moderate effect size (*d* = 0.65) and a similar cut-off score (12.88 sec; AUC value = 0.70) (10). Sharma et al. (14) contrasted SHT performance between functional ankle instability participants with giving way (FAI-GW), FAI without giving way (FAI-NGW) and a control group (14). They found significant differences between both FAI groups and the control group (14). During jumping and landing, the active and passive stabilisers of the ankle joint are challenged by excessive ankle supination and pronation movement (10). This movement is similar to LAS incidence, and participants in previous studies reported an unstable sensation in their ankle during the SHT (14, 19). In this study, individuals in the positive SLBT group had lower CAIT scores for the CAI limb (*P* < 0.001), which may explain why their self-reported feeling of ankle joint instability was higher. Although both groups reported similar frequencies of feeling ankle joint instability in the past 6 months (*P* = 0.082), this data might explain the positive SLBT group’s worse results on the SHT.

Both dynamic balance measures, the mSEBT-PM and SHT, are related to sport movements, such as hopping, cutting and squatting. Hence, we recommend that clinicians selectively apply these tests with their respective cut-off points to suit each individual’s sport of choice. For instance, the SHT could be prioritised in basketball and soccer players.

This study has some limitations. First, the baseline mean CAIT scores for both groups are different, and it is possible that the CAIT scores confounded the balance differences among groups. Unfortunately, there is no standard criterion to determine a CAIT score that accounts for the severity of balance impairment in the CAI population. Because all participants had CAIT scores ≤ 25 on the CAI limb, we attempted to control other factors to enhance the homogeneity of both groups. Namely, we matched participants based on their age, sex, weight, height and CAI limb (10). To improve the methodology, future studies might establish a criterion for CAIT scores to classify balance impairment severity among CAI participants. Second, our study is only the first step towards a better understanding of how these clinical tests can be used to efficiently identify balance impairment. Future studies, such as a prospective study that applied treatment relative to these cut-off scores, could improve clinical benefits. Third, most of our participants were male (20 per group) and the generalisability of our cut-off scores to female populations should be considered with caution. Thus, future studies should recruit more female athletes and establish cut-off scores specifically for females. Fourth, this study was conducted in a university-based setting and the present cut-off scores mainly apply to young amateur athletes. Future studies might recruit wider populations, such as elite athletes, high school athletes or participants with a greater age range, to enhance the generalisability of the cut-off scores.

## Conclusion

The present study presents diagnostic values that could be utilised in clinical diagnosis. Healthcare professionals and athletic trainers can gain benefits by recognising these cut-off scores to quantify and diagnose balance impairment among individuals with CAI. To improve the clinical benefits and meaningfulness of these tests, further research should apply these specific cut-off scores as indicators of injury risk or as monitoring tools after rehabilitation.

## Figures and Tables

**Table 1:** Characteristics of CAI participants in negative and positive SLBT groups

Variables	Group, mean ± SD		*P*-value

	Negative SLBT group (*n* = 27)	Positive SLBT group (*n* = 27)	
CAI limb (right/left)^a^	15/12	15/12	–
Age (years)	23.29 ± 5.13	22.62 ± 4.84	0.627
Height (cm)	171.0 ± 0.06	171.0 ± 0.08	0.899
Weight (kg)	67.03 ± 8.23	67.71±12.44	0.813
CAIT scores (scores)			
• CAI limb	18.92 ± 3.53	16.11 ± 4.71	0.015*
• Contralateral limb	25.11 ± 4.89	24.48 ± 5.01	0.629
History of LAS			
• Number of LAS (numbers)	5.52 ± 3.84	6.18 ± 4.05	0.538
• Duration since the first LAS (months)	76.11 ± 62.11	74.60 ± 55.53	0.935
• Duration since the latest LAS (months)	11.33 ± 14.08	18.17 ± 22.18	0.185
Giving way in past 6 months (numbers)	4.44 ± 4.73	5.65 ± 4.58	0.348
Feeling of ankle joint instability in past 6 months (numbers)	4.92 ± 3.91	7.30 ± 5.77	0.082

Notes: *P*-value of independent sample *t*-test;

a numbers in each limb;

* statistical significant difference between groups

**Table 2:** Comparison of balance performance between CAI individuals in negative and positive SLBT groups

Variables	Group, mean ± SD		Mean differences (95% CI)	Effect sizes	*P*-value

	Negative SLBT group (*n* = 27)	Positive SLBT group (*n* = 27)			
FLT (number of foot lifts)	7.38 ± 4.45	13.33 ± 5.81	−5.95 (−8.77, −3.12)	1.16	< 0.001*
TIBT (seconds)	49.00 ± 15.10	16.76 ± 11.15	32.24 (24.98, 39.48)	2.45	< 0.001*
mSEBT-PM (normalised reaching distances)	93.08 ± 6.79	83.20 ± 10.07	9.00 (5.00, 14.00)	1.22	< 0.001*
SHT (seconds)	11.62 ± 4.37	21.10 ± 13.43	−9.48 (−14.93, −4.02)	1.06	< 0.001*

Notes: Effect sizes were demonstrated as (Cohen’s *d*); *P*-value = *P*-value of independent sample *t*-test;

* statistical significant difference between groups

**Table 3:** Discriminative validity of clinical tests between CAI participants in negative and positive SLBT groups

Tests	Cut-off scores	AUC values (95% Confidence significance interval)	Asymptotic	Sensitivity	Specificity	Youden index (*J*)	LR+	LR−
FLT	9.50 number of foot lifts	0.80 (0.673–0.911)	< 0.001*	0.74	0.71	45	2.55	0.36
TIBT	30.40 sec	0.94 (0.890–0.998)	< 0.001*	0.93	0.82	75	5.16	0.08
mSEBT-PM	91.05 normalised reaching distances	0.80 (0.690–0.926)	< 0.001*	0.78	0.70	48	2.60	0.31
SHT	12.85 sec	0.80 (0.695–0.923)	< 0.001*	0.74	0.71	45	2.55	0.36

Notes: LR+ = positive likelihood ratio; LR− = negative likelihood ratio;

* statistical significant difference between groups

## References

[1] Kobayashi T, Gamada K (2014). Lateral ankle sprain and chronic ankle instability: a critical review. Foot Ankle Spec.

[2] Gribble PA, Bleakly CM, Caulfield BM, Docherty CL, Fourchet F, Fong DT (2016). Evidence review for the 2016 international ankle consortium consensus statement on the prevalence, impact and long-term consequences of lateral ankle sprains. Br J Sports Med.

[3] Hertel J, Corbett RO (2019). An updated model of chronic ankle instability. J Athl Train.

[4] Gribble PA, Delahunt E, Bleakley C, Caufield B, Docherty C, Fourchet F (2013). Selection criteria for patients with chronic ankle instability in controlled research: a position statement of the International Ankle Consortium. Br J Sports Med.

[5] Arnold BL, Motte SDL, Linens SW, Ross SE (2009). Ankle instability is associated with balance impairments: a meta-analysis. Med Sci Sports Exerc.

[6] KOJ, Rosen AB, Brown CN (2017). Comparison between single and combined clinical postural stability tests in individuals with and without chronic ankle instability. Clin J Sport Med.

[7] Wikstrom EA, Brown CN (2014). Minimal reporting standards for copers in chronic ankle instability research. Sport Med.

[8] Thompson C, Schabrun S, Romero R, Bialocerkowski A, Dieen JV, Marshall P (2018). Factors contributing to chronic ankle instability: a systematic review and meta-analysis of systematic reviews. Sport Med.

[9] Trojian TH, McKeag DB (2006). Single leg balance test to identify risk of ankle sprains. Br J Sports Med.

[10] Linens SW, Ross SE, Arnold BL, Gayle R, Pidcoe P (2014). Postural-stability tests that identify individuals with chronic ankle instability. J Athl Train.

[11] Hiller CE, Refshauge KM, Herbert RD, Kilbreath SL (2007). Balance and recovery from a perturbation are impaired in people with functional ankle instability. Clin J Sport Med.

[12] Chrintz H, Falster O, Roed J (1991). Single-leg postural equilibrium test. Scand J Med Sci Sports.

[13] Hertel J, Braham RA, Hale SA, Olmsted-Kramer LC (2006). Simplifying the star excursion balance test: analyses of subjects with and without chronic ankle instability. J Orthop Sports Phys Ther.

[14] Sharma N, Sharma A, Sandhu JS (2011). Functional performance testing in athletes with functional ankle instability. Asian J Sports Med.

[15] Norman GR, Streiner DL (2008). Analysis of variance: comparing two groups. Biostatistics: the bare essentials.

[16] Wright CJ, Arnold BL, Ross SE, Linens SW (2014). Recalibration and validation of the cumberland ankle instability tool cutoff score for individuals with chronic ankle instability. Arch Phys Med Rehabil.

[17] Hiller CE, Refshauge KM, Bundy AC, Herbert RD, Kilbreath SL (2006). The cumberland ankle instability tool: a report of validity and reliability testing. Arch Phys Med Rehabil.

[18] Gribble PA, Hertel J, Plisky P (2012). Using the star excursion balance test to assess dynamic postural-control deficits and outcomes in lower extremity injury: a literature and systematic review. J Athl Train.

[19] Docherty CL, Arnold BL, Gansneder BM, Hurwitz S, Gieck J (2005). Functional-performance deficits in volunteers with functional ankle instability. J Athl Train.

[20] Kim KT (2015). *t*-Test as a parametric statistic. Korean J Anesthesiol.

[21] Fritz C, Morris PE, Richler JJ (2011). Effect size estimates: current use, calculations, and interpretation. J Exp Psy: Gen.

[22] Hearst M, Kohn MA, Lo B, Novotny TE (2013). Designing clinical research.

[23] Wikstrom EA, Tillman MD, Chmielewski TL, Cauraugh JH, Naugle KE, Borsa PA (2012). Discriminating between copers and people with chronic ankle instability. J Athl Train.

[24] Rosen AB, Needle AR, Ko J (2019). Ability of functional performance tests to identify individuals with chronic ankle instability: a systematic review with meta-analysis. Clin J Sport Med.

[25] Hertel J (2002). Functional anatomy, pathobiomechanics and pathophysiology of lateral ankle instability. J Athl Train.

[26] Munn J, Sullivan SJ, Schneiders AG (2010). Evidence of sensorimotor deficits in functional ankle instability: a systematic review with meta-analysis. J Sci Med Sport.

[27] Plisky PJ, Rauh MJ, Kaminski TW, Underwood FB (2006). Star excursion balance test as a predictor of lower extremity injury in high school basketball players. J Orthop Sports Phys Ther.

[28] Plante JE, Wikstrom EA (2013). Differences in clinician-oriented outcomes among controls, copers, and chronic ankle instability groups. Phys Ther Sport.

